# Efficacy of functional electrical stimulation alone and as an adjunct to exercise for improving respiratory function and aerobic capacity in spinal cord injury: a systematic review and meta-analysis

**DOI:** 10.3389/fresc.2025.1623752

**Published:** 2025-07-31

**Authors:** Jiahao Xiangli, Binquan Ma, Yu Liang, Shi Haijiangshi, Xifang Liu

**Affiliations:** ^1^ Department of Rehabilitation, Honghui Hospital, Xi'an Jiaotong University, Xi'an, Shaanxi, China; ^2^ Department of Sport and Health Sciences, Xi'an Physical Education University, Xi'an, Shaanxi, China; ^3^ The Key Laboratory of Biomedical Information Engineering of Ministry of Education, Xi'an Jiaotong University, Xi'an, Shaanxi, China

**Keywords:** functional electrical stimulation, spinal cord injury, respiratory rehabilitation, physical agents therapy, exercise therapy

## Abstract

**Objective:**

To systematically evaluate the efficacy of functional electrical stimulation (FES), used either alone or as an adjunct to exercise (rowing/cycling), for improving respiratory function and aerobic capacity in patients with spinal cord injury (SCI).

**Methods:**

We conducted a PRISMA-compliant meta-analysis, searching PubMed, Embase, Cochrane Library, and Web of Science through January 2025. We included 23 randomized controlled trials and self-controlled studies (*N* = 314) that assessed outcomes such as forced vital capacity (FVC), peak expiratory flow (PEF), maximal expiratory/inspiratory pressure (MEP/MIP), and peak oxygen uptake (VO₂peak).

**Results:**

When used as a standalone intervention, FES significantly improved expiratory function, with notable increases in PEF (SMD = 0.42, *p* = 0.007), MEP (SMD=0.93, *p* = 0.008), and FVC (SMD = 0.37, *p* = 0.03). However, no significant improvement was found for MIP (*p* = 0.38). When FES was combined with exercise, it significantly enhanced aerobic capacity. This was demonstrated by improvements in VO₂peak for both FES-assisted rowing (SMD = 0.35, *p* = 0.03) and FES-assisted cycling (SMD = 0.24, *p* = 0.0003) compared to exercise alone. No significant effects on peak ventilation were observed.

**Conclusion:**

FES moderately improves key expiratory functions in individuals with SCI and acts synergistically with exercise to augment aerobic capacity. These findings support the clinical use of FES in this population. However, the interpretation of these results should consider the methodological heterogeneity across studies and the limited sample size for some outcomes.

**Systematic Review Registration:**

https://www.crd.york.ac.uk/PROSPERO/myprospero, identifier CRD420251030235.

## 1 Introduction

Spinal cord injury (SCI) affects 1.3 million North Americans, with traumatic injuries accounting for over half of these cases. Data from the National Spinal Cord Injury Statistical Center (2017) indicate that traumatic SCI occurs at a rate of 54 per million population annually in the U.S., translating to 17,500 new diagnoses yearly. Cumulatively, this condition impacts an estimated 285,000 Americans, with prevalence ranging between 245,000 and 345,000 cases (1, 2). Cervical SCI often leads totetraplegia, resulting in varying degrees of paralysis and sensory loss in all four limbs and the trunk, depending on the injury's level and severity. This paralysis often involves the primary respiratory muscles: the diaphragm, intercostals, and abdominal muscles. Typically, individuals with motorcomplete injuries below C3 retain some diaphragmatic function, allowing for unassisted breathing. However, paralysis of the intercostal and abdominal muscles reduces ventilatory capacity, a leading contributor to rehospitalization due to respiratory complications (3). Furthermore, thoracic SCI can impair accessory respiratory muscle function. While these muscles normally coordinate with the diaphragm, SCI-related respiratory muscle paralysis or increased oxygen demand can decrease aerobic capacity during exercise, leading to dyspnea during daily activities (4).

**Table 1:** Overview of included indicators and measurement standards.

Domain	Parameters	Measurement Standards
Lung volumes	FVC	Spirometry (ATS/ERS guidelines)
Airflow	PEF	Peak flow meter (best of 3 attempts)
Muscle strength	MIP, MEP	Digital manometer (Valsalva/Mueller maneuvers)
Aerobic capacity	VO₂peak, VE	Cardiopulmonary exercise testing

Pulmonary function assessment relies on key spirometric parameters: forced vital capacity (FVC, total air exhaled after maximal inhalation), peak expiratory flow (PEF, maximum exhalation velocity), maximal expiratory pressure (MEP, highest expiratory force), and maximal inspiratory pressure (MIP, peak inspiratory effort). These values are significantly reduced in individuals with SCI-induced tetraplegia compared to healthy populations (2, 4). It is well-established that spinal cord injury (SCI) directly compromises respiratory muscle strength and pulmonary function, manifesting as significant reductions in forced vital capacity (FVC), maximal inspiratory pressure (MIP), and maximal expiratory pressure (MEP) (3). Minute ventilation (VE) reflects the matching of alveolar ventilation and pulmonary perfusion (5), while peak oxygen uptake (VO2peak) indicates the patient's cardiopulmonary capacity and the effectiveness of training interventions (6).

Functional electrical stimulation (FES) induces muscle contraction by electrically stimulating motor nerves. Research suggests that FES of the abdominal muscles can improve respiratory function in individuals with SCI. The proposed mechanism involves FES-induced abdominal muscle contraction, leading to increased intra-abdominal pressure, enhanced expiratory pressure, and ultimately, improved expiratory flow. This process also reduces total lung capacity below functional residual capacity, and the subsequent passive recoil during inspiration increases tidal volume (7, 8). Current therapeutic interventions for respiratory dysfunction in spinal cord injury (SCI) patients encompass respiratory muscle training (9), progressive resistance training (10), mechanical ventilation (11), diaphragmatic pacing (13), and transcranial magnetic stimulation (TMS) (12). Conventional therapeutic regimens predominantly rely on respiratory muscle training and progressive resistance training, supplemented by mechanical ventilation (11) and diaphragmatic pacing (13). While respiratory muscle training and progressive resistance training have demonstrated well-established efficacy in respiratory function improvement (9, 10), their therapeutic effectiveness is contingent upon active patient participation. Mechanical ventilation may exert detrimental effects on diaphragmatic function (11), whereas diaphragmatic pacing remains underutilized due to multiple medical constraints including high surgical implantation costs, prolonged rehabilitation phases, and potential phrenic nerve complications (13). Recent investigations suggest that although TMS exhibits therapeutic promise, its precise mechanisms of action require further elucidation, with current research primarily confined to animal models (12). In contrast, functional electrical stimulation (FES) emerges as a superior passive therapeutic modality (14). Particularly advantageous for critically ill patients or those with limited treatment compliance, FES circumvents diaphragmatic functional impairment associated with mechanical ventilation Notably, compared to TMS, FES operates through more clearly defined mechanisms and has demonstrated clinically validated therapeutic benefits in SCI populations. While McCaughey et al. (2016) (8) explored FES efficacy in SCI, their analysis was limited to PubMed and lacked subgroup analyses on adjunctive exercise types (15). Consequently, although these investigations yield preliminary evidence regarding FES-mediated respiratory modulation in SCI, methodological comprehensiveness remains circumscribed by analytical scope limitations necessitating further epidemiological corroboration. This quantitative evidence synthesis investigates the therapeutic efficacy of transabdominal functional electrical stimulation (FES) in enhancing pulmonary performance parameters among spinal cord injury (SCI) populations, while concurrently evaluating the cardiorespiratory optimization potential of FES combined with adjunctive exercise modalities. The growing interest in adjunctive modalities like FES, there remains limited consensus on its efficacy in improving pulmonary function in SCI patients. This study aims to systematically evaluate the impact of FES on respiratory outcomes in this population.

## 2 Data and metho

### 2.1 Literature search strategy

(a) Searchers: Two independent researchers conducted the literature searches according to the established search strategy, without interference. A third researcher resolved any disagreements.
(b) Databases: The following English databases were searched: PubMed, Embase, The Cochrane Library, and Web of Science. All databases utilized Medical Subject Headings (MeSH) terms with Boolean operators to optimize sensitivity
(c) Search Terms: The following search terms were used in the English databases: (1) MeSH terms Electric Stimulation Therapy"[Mesh]" (2) Spinal Cord Injuries"[Mesh]" (3) Respiratory Function Tests"[Mesh] (4) Free-text keywords (title/abstract):"FES” OR “functional electrical stimulation” “spinal cord injury” OR “SCI” “respiratory dysfunction” OR “pulmonary function”
(d) Search Timeframe: Limited to English studies due to translational capacity constraints. Inception to 17 January 2025.
  Screening Platform: EndNote 21 was used for deduplication and blinded title/abstract screening, with exclusion reasons documented in PRISMA flow diagrams.
(e) Literature Search Strategy: A combination of subject headings (MeSH terms) and keywords was used for the search.

The protocol for this systematic review was prospectively registered with the International Prospective Register of Systematic Reviews (PROSPERO) on 10 April 2025 (Registration ID: CRD420251030235). The full registration details are publicly accessible through the PROSPERO database at https://www.crd.york.ac.uk/PROSPERO/myprospero (accessed during the protocol development phase).

### 2.2 Inclusion criteria and exclusionary determinants

#### 2.2.1 Inclusion criteria

(1) Specifically: (a) prospectively randomized trials employing within-cohort comparative designs (experimental arms as internal controls), and (b) self-referential trial architectures adhering to epidemiological validation standards.
(2) Study Population: Individuals with spinal cord injury and respiratory dysfunction.
(3) Intervention: Functional electrical stimulation (FES) was used as the primary intervention, with pre- and post-FES baseline measurements serving as the patient's own control.
(4) Inclusion criteria mandated the inclusion of one or more key respiratory parameters: Eligible studies for this meta-analysis included those investigating individuals with spinal cord injury (SCI). While no specific age restrictions were applied for participant inclusion (i.e., studies on both adults and children would have been considered eligible if they met other criteria), all ultimately included studies focused on adult populations. Respiratory dysfunction, as a characteristic for study inclusion or as an outcome of interest, was defined if primary studies reported one or more of the following: (a) Forced Vital Capacity (FVC) < 80% of the predicted value, (b) Maximal Inspiratory Pressure (MIP) < 60 cmH₂O, or (c) explicit documentation of respiratory dysfunction by the original study authors. FVC (forced vital capacity), PEF measurements (peak expiratory flow), MEP/MIP values (maximal expiratory/inspiratory pressure), ventilation volume (VE), and VO2peak (peak oxygen uptake).

These indicators were selected because FVC reflects the maximum volume of air that can be forcibly exhaled. PEF reflects the peak expiratory flow rate, representing the maximum instantaneous expiratory flow generated during a forceful and rapid exhalation (16). MIP and MEP reflect the strength of the inspiratory and expiratory muscles, respectively (17). These respiratory physiological indicators collectively constitute a multidimensional evaluation framework for pulmonary physiological status, involving ventilatory volume dynamics. Ventilatory volume dynamics (VE) serve as biomarkers delineating the pathophysiological coupling between alveolar gas exchange and capillary perfusion (5), whereas maximal aerobic capacity metrics (VO2peak) operationalizes cardiorespiratory functional reserves as a benchmark for therapeutic regimen efficacy quantification (6). These two indicators reflect the patient's aerobic capacity and assess the impact of training.

#### 2.2.2 Exclusion criteria

Studies were excluded if they met any of the following criteria:

(1) Lacked essential data required for meta-analysis (e.g., missing mean, standard deviation, or sample size for key outcomes).
(2) Were reviews, conference abstracts/papers, letters to the editor, case reports, or other meta-analyses.
(3) Represented duplicate publications of the same study cohort and data (in such cases, the most comprehensive or most recent publication was retained).
(4) Exhibited critical methodological limitations or a high/critical risk of bias that could substantially compromise the validity of their findings. The assessment of methodological quality and risk of bias was conducted as follows:

For non-randomized studies of interventions (NRSI), bias risk quantification was performed using the Cochrane ROBINS-I (Risk Of Bias In Non-randomized Studies—of Interventions) tool. Studies rated as having a "Critical" overall risk of bias, or a "High" risk of bias in domains crucial to the interpretation of the primary outcomes of this meta-analysis, were excluded.

For quasi-experimental studies (where ROBINS-I might not be fully applicable for all aspects or to complement its findings), methodological rigor was further assessed using the Joanna Briggs Institute (JBI) Critical Appraisal Checklist for Quasi-Experimental Studies (non-randomized experimental studies). Studies failing to meet a predefined threshold of essential quality criteria (e.g., demonstrating major flaws in several key areas such as comparability of groups at baseline if applicable, clear description of the intervention, reliable outcome measurement, and appropriate statistical analysis) that would lead to a judgment of low overall methodological quality, were excluded.

### 2.3 Data acquisition protocol

Two reviewers independently extracted data using a piloted Excel template adapted from DECiMAL guidelines. Extracted variables included:

(1) Study ID (author/year)
(2) Participant demographics (age, SCI level, injury duration)
(3) FES protocol (frequency, intensity, duration)
(4) Respiratory/aerobic outcomes
(5) Discrepancies were resolved *via* consensus or third reviewer arbitration (*κ* = 0.81).

### 2.4 Assessment of risk of bias in included studies

Given the predominance of intra-participant comparator frameworks within the trial architectures, bias risk quantification was conducted through the Cochrane ROBINS-I instrument for non-randomized studies, while methodological rigor assessment leveraged the Joanna Briggs Institute (JBI) Critical Appraisal Checklist for quasi-experimental investigations. Key appraisal criteria were used to assign quality ratings and scores to each study.

The Cochrane ROBINS-I instrument systematically evaluates methodological distortions across seven critical dimensions:

(1) Uncontrolled confounding variables
(2) Participant selection criteria misalignment
(3) Interventional categorization inaccuracies
(4) Protocol adherence deviations
(5) Incomplete data artifacts
(6) Outcome measurement inconsistencies
(7) Selective reporting tendencies.

Methodologists systematically classified each dimension using tripartite risk stratification: “controlled distortion,” “substantial distortion,” and “indeterminate distortion” through consensual expert evaluation.

The JBI critical appraisal checklist includes the following criteria:

1. Appropriateness of study design
2. Similarity of intervention and control groups
3. Consistency of outcome measurement
4. Control of confounding factors
5. Data integrity
6. Appropriateness of statistical analysis
7. Reasonableness of results interpretation.

**Figure 1:**
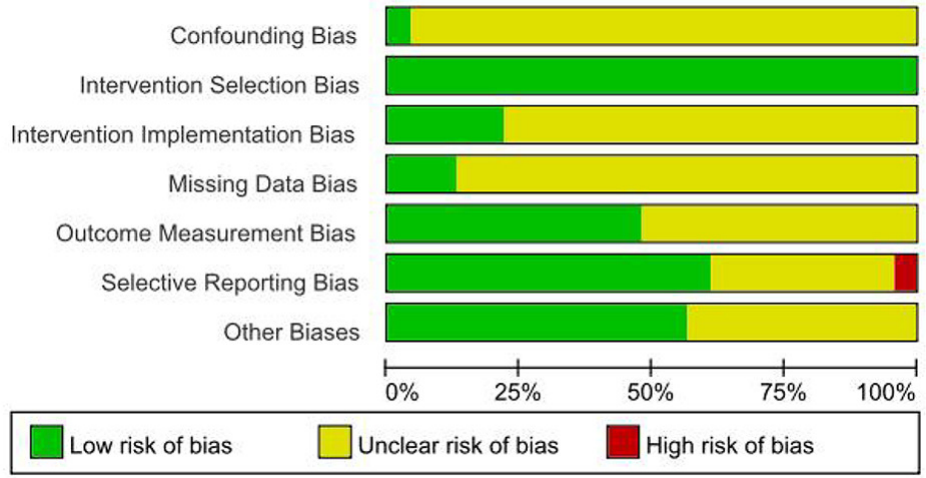
Quality evaluation results of the included literature. Figure Legend: Low risk of bias, Unclear risk of bias, High risk of bias.

**Figure 2:**
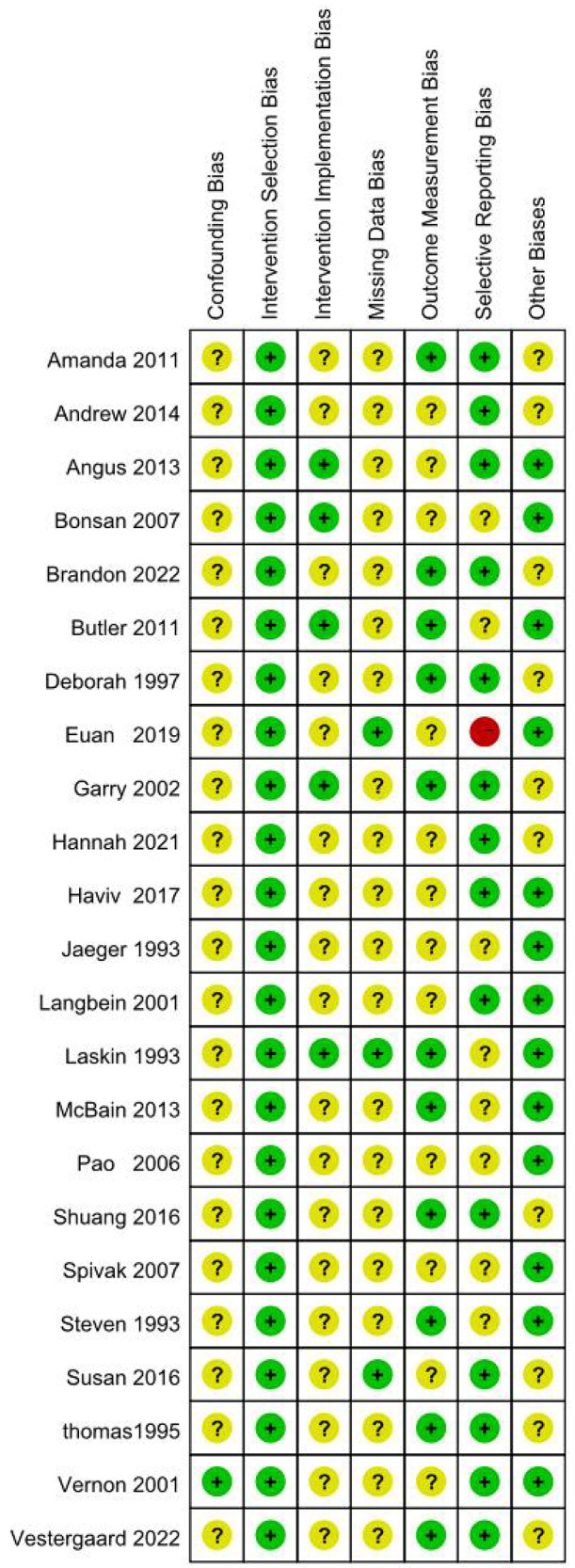
Evaluation summary of the included literature. Figure note: “-” = high risk, “?” = unclear risk, “+” = low risk.

Two researchers independently performed the appraisal, with disagreements resolved through consultation with a third researcher. Results were visualized in heatmaps (Figures 1, 2) and incorporated into GRADE evidence profiles.

### 2.5 Outcome measures

This investigation quantified core pulmonary responses to functional electrical stimulation (FES) in spinal cord injury patients through four principal biomarkers: forced vital capacity (FVC), peak expiratory flow (PEF), and maximum pressure thresholds during expiration/inspiration (MEP/MIP).Aerobic capacity parameters, including peak ventilation (VE) and peak oxygen uptake (VO2peak), were also assessed to determine the impact of combined exercise interventions (Table 1).

### 2.6 Statistical inference framework

Evidence synthesis was operationalized through Cochrane Review Manager 5.4 (RRID: SCR_003581) under continuous measurement paradigms, with all endpoints undergoing interval-scale normalization. Restricted maximum likelihood (REML) estimation procedures were implemented for between-study variance components, accompanied by prediction interval construction for clinical interpretability enhancement. When studies used the same measurement methods and units, the mean difference (MD) and 95% confidence interval (CI) were used as the effect size. When measurement methods or units differed, the standardized mean difference (SMD) and 95% CI were used. Heterogeneity among studies was assessed using the *χ*2 test (significance level *α* = 0.1). A fixed-effects model was used when *P* ≥ 0.1 and *I*^2^ < 50%, indicating low heterogeneity. A random-effects model was used when *P* < 0.1 and *I*^2^ ≥ 50%, Continuous outcomes: Standardized mean difference (SMD) with 95% CI using:Fixed-effects model (*I*^2^ < 50%, *p* ≥ 0.1)Random-effects model (*I*^2^ ≥ 50%, *p* < 0.1) Sensitivity analyses excluded studies with high ROBINS-I bias Publication bias: Egger's test (*p* < 0.05 considered significant) Sensitivity analyses were conducted to assess the robustness of the pooled estimates. These included: (a) Leave-one-out validation: Systematically excluding one study at a time to quantify its influence on the composite effect magnitude. (b) Stratified sensitivity analysis based on methodological quality: Studies were stratified based on their risk of bias assessment (e.g., “low risk of bias” vs. “moderate/high/critical risk of bias” as determined by ROBINS-I and/or JBI appraisals). The pooled effect sizes were then re-calculated for subgroups of higher-quality studies, or by excluding studies with a high risk of bias/low methodological quality, to ascertain the impact of study quality on the overall findings. Indicating substantial heterogeneity. Stratified covariate interrogation was conducted through Bayesian multilevel modeling to identify moderator variables contributing to inter-study variance. Robustness validation employed iterative leave-one-out validation protocols, systematically excluding singular datasets to quantify their influence on composite effect magnitude. For outcomes demonstrating significant dispersion metrics (*I*^2^ ≥ 50%), publication bias diagnostics were implemented via radial regression asymmetry profiling (Egger's test equivalence), with all inferential analyses maintaining Bonferroni-adjusted α thresholds of 0.05 for two-tailed hypothesis testing.

To address potential ambiguity, it is noted that control groups in the included primary studies varied. For analyses of “FES alone,” the comparators (termed “non-FES” in forest plots) included sham FES, no intervention, or standard care without FES. For analyses of “FES combined with exercise,” the comparators (termed “non-FES exercise” or similar) consisted of the equivalent exercise modality (e.g., rowing, cycling) performed without FES.

## 3 Results

### 3.1 Literature search and selection

**Figure 3:**
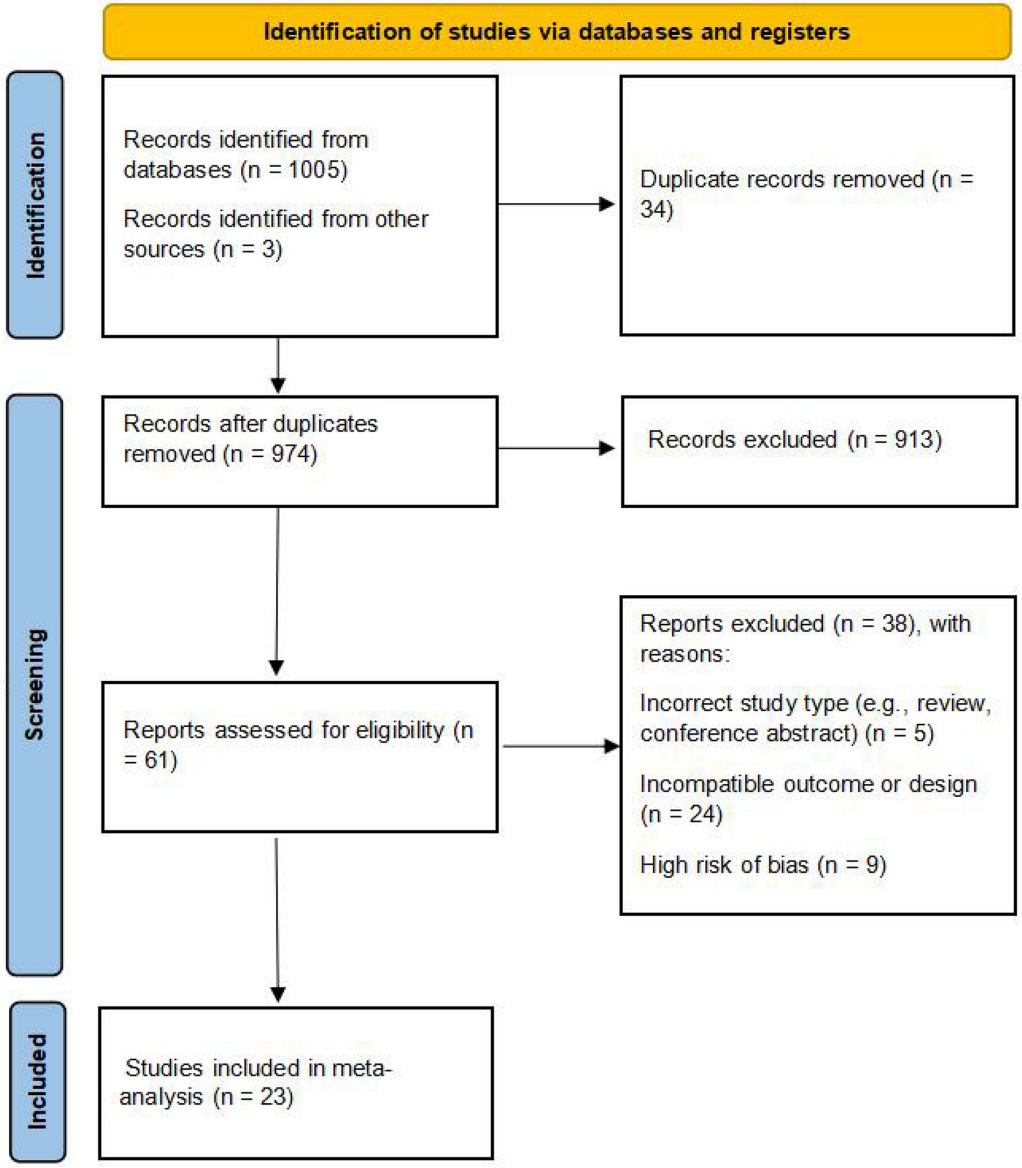
Flow chart of literature screening.

The initial search identified 1005 articles. After removing duplicates using EndNote 21 and screening titles and abstracts for relevance, 29 articles were considered potentially eligible. Following full-text review, 23 articles met the inclusion criteria and were included in the meta-analysis (Figure 3).

### 3.2 Characteristics of included studies

The final analysis comprised 23 articles, with 3 identified through supplementary searches. Twelve studies investigated the effect of functional electrical stimulation (FES) on respiratory function in individuals with spinal cord injury (SCI) (8, 13, 18–26, 41), while 11 examined the effect of FES combined with exercise on aerobic capacity (27–36). Among the 12 exclusive FES trials, outcome documentation frequencies were distributed as: 8 investigations quantified peak airflow rates (PEF), 5 assessed expiratory force thresholds (MEP), 4 evaluated forced breathing capacity (FVC), and 2 measured inspiratory pressure limits (MIP). The 11 exercise-combined FES protocols incorporated rowing-based (*n* = 7) and cycling-focused (*n* = 4) interventions. A subset of six rowing investigations concurrently measured ventilatory output maxima (VE) and maximal aerobic capacity (VO2peak).Outcome reporting was less consistent for cycling, with 2 studies reporting VO2peak and 2 reporting VE. Detailed characteristics of the included studies are presented in Tables 2, 3.

**Table 2:** Basic characteristics of the included literature.

Study	Article Quality	Region	Study Type	No. (Total Participants)	Age (mean ± SD)	Gender (Male, Female)
McCaughey et al., (8)	Medium	Australia	Randomized Controlled Trial	20 (actual 14)	58.8 ± 13.09	12, 8
McLachlan et al., (19)	Medium	UK	Self-controlled	12	31 (mean)	Not reported
Cheng et al., (18)	Medium	Taiwan	Randomized Controlled Trial	26	36.75 ± 13.64	5, 21
Linder (20)	Medium	USA	Controlled	11	37.45 ± 10.11	11, 0
Lin et al., (26)	Medium	USA	Self-controlled	8	51 ± 8	Not reported
Lee et al., (21)	Medium	Australia	Single Case	1	65	1, 0
Jaeger et al., (37)	Medium	USA	Comparative Experimental	24 (actual 14)	34 ± 11.5	5, 19
Langbein et al., (22)	Medium	USA	Self-controlled Experimental	10	50.4 ± 14.0	10, 0
McBain et al., (23)	Medium	Australia	Randomized Crossover	15	45 ± 15.5	15, 0
Butler et al., (38)	Medium	Australia	Experimental Self-controlled	11	45 ± 16.58	7, 4
Spivak et al., (24)	Medium	Israel	Self-controlled	10	40.1 ± 13.27	10, 0
Haviv et al., (25)	Medium	Israel	Self-controlled	14	39.64 ± 11.2	12, 4
Mercier et al., (27)	Medium	USA	Self-controlled	27	29.8 ± 6.2	26, 1
Taylor et al., (28)	Medium	France	Randomized Controlled Trial	14	39.2 ± 3.3	13, 1
Yates et al., (29)	Medium	USA	Randomized Controlled Trial	21	29 ± 6	18, 3
Qiu et al., (30)	Medium	USA	Self-controlled	12	33.3 ± 3.8	12, 0
Vestergaard et al., (36)	Medium	Denmark	Self-controlled	8	42.75 ± 14.61	7, 1
Carty et al., (40)	Medium	Ireland	Prospective Cohort	14	45.08 ± 7.92	11, 3
Barstow et al., (34)	Medium	UK	Self-controlled	6	35.8 ± 5.0	Not reported
Mutton et al., (35)	Medium	USA	Non-randomized Controlled Trial	11	35.6 ± 6.6	Not reported
Wilbanks et al., (31)	Medium	USA	Self-controlled	10	47.2 ± 18.3	8, 2
Wheeler et al., (32)	Medium	USA	Quasi-experimental	6	42.5 ± 17.9	Not reported
Laskin et al., (33)	Medium	Canada	Self-controlled	8	27.87 ± 4.16	7, 1

**Table 3:** Characteristics of interventions and outcomes of the included literature.

Study	Quality	Intervention	Treatment Protocol	Stimulation Parameters	Outcome Measures	Adverse Effects
McCaughey et al., (8)	Medium	FES	Experimental: FES vs. Sham FES, 5x/week, 2x/day until discharge	30 Hz, 350 μs	④⑤	NR
McLachlan et al., (19)	Medium	FES	Pre-post FES, 4x/week, 20–60 min/session ×4 weeks	30 Hz, 50 μs	①③	NR
Cheng et al., (18)	Medium	FES	Experimental: FES vs. No FES, 5x/week, 30 min/day ×4 weeks	30 Hz, 300 ms	①②③④⑤	NR
Linder (20)	Medium	FES	3 groups (Group 3 FES), details NR	50 Hz, 300 μs	④	NR
Lin et al., (26)	Medium	FES	Pre-post FES, 5x/week, 30 min/session ×4 weeks	20 Hz, 2 sec bursts	④	NR
Lee et al., (21)	Medium	FES	Pre-post FES ×4 weeks	50 Hz, 200 μs	④	NR
Jaeger et al., (37)	Medium	FES	Manual-assisted FES, pre-post, details NR	50 Hz, 0.3 ms	③	NR
Langbein et al., (22)	Medium	FES	Single session pre-post	50 Hz, 250 ms	①②③	NR
McBain et al., (23)	Medium	FES	Crossover, 5x/week	50 Hz	①②③	NR
Butler et al., (38)	Medium	FES	Pre-post, details NR	50 Hz, 200 μs	②③	NR
Spivak et al., (24)	Medium	FES	Manual group, details NR	0.3 ms	③	NR
Haviv et al., (25)	Medium	FES	Pre-post, details NR	100 Hz, 200 μs	③	NR
Mercier et al., (27)	Medium	FES + Rowing	Pre-post, 3x/week, 20 min ×6 months	40 Hz, 300 ms	⑥⑦	NR
Taylor et al., (28)	Medium	FES + Rowing	Pre-post, 3x/week, 30 min ×4 weeks	40 Hz, 450 ms	⑥⑦	NR
Yates et al., (29)	Medium	FES + Rowing	Pre-post, 3x/week, 30 min ×12 weeks	NR	⑥⑦	NR
Qiu et al., (30)	Medium	FES + Rowing	Pre-post, 3x/week, 30 min ×6 months	NR	⑥⑦	NR
Wilbanks et al., (31)	Medium	FES + Rowing	Pre-post, 3x/week, 30 min ×6 weeks	NR	⑥⑦	NR
Wheeler et al., (32)	Medium	FES + Rowing	Pre-post, details NR	NR	⑥	NR
Laskin et al., (33)	Medium	FES + Rowing	Pre-post, details NR	NR	⑦	NR
Vestergaard et al., (36)	Medium	FES + Cycling	3 groups (Group 1)	50–500 μs	⑦	NR
Carty et al., (40)	Medium	FES + Cycling	Pre-post, 5x/week, 60 min ×8 weeks	NR	⑦	NR
Barstow et al., (34)	Medium	FES + Cycling	Details NR	30 Hz	⑥	NR
Mutton et al., (35)	Medium	FES + Cycling	Pre-post (Phase 1), details NR	30 Hz	⑥	NR

NOTE: ① FVC, ② FEV1, ③ PEFR, ④ MEP, ⑤ MIP, ⑥ VEpeak, ⑦ VO₂peak; NR = Not Reported.

### 3.3 Quality assessment of included studies

The initial search identified 1,005 articles. After removing duplicates using EndNote 21 and screening titles and abstracts for relevance, 29 articles underwent full-text assessment. After comprehensive full-text appraisal, 9 manuscripts were disqualified during quality triage, retaining 20 investigations meeting phase I eligibility thresholds. *Post hoc* incorporation of 3 supplemental datasets was achieved through supplementary retrieval protocols (including citation tracking and grey literature mining), culminating in 23 methodologically validated studies for quantitative synthesis, as delineated in the PRISMA flow diagram (Figure 3).

### 3.4 Meta-analysis results

#### 3.4.1 Effect of FES on respiratory function in SCI

**Figure 4:**
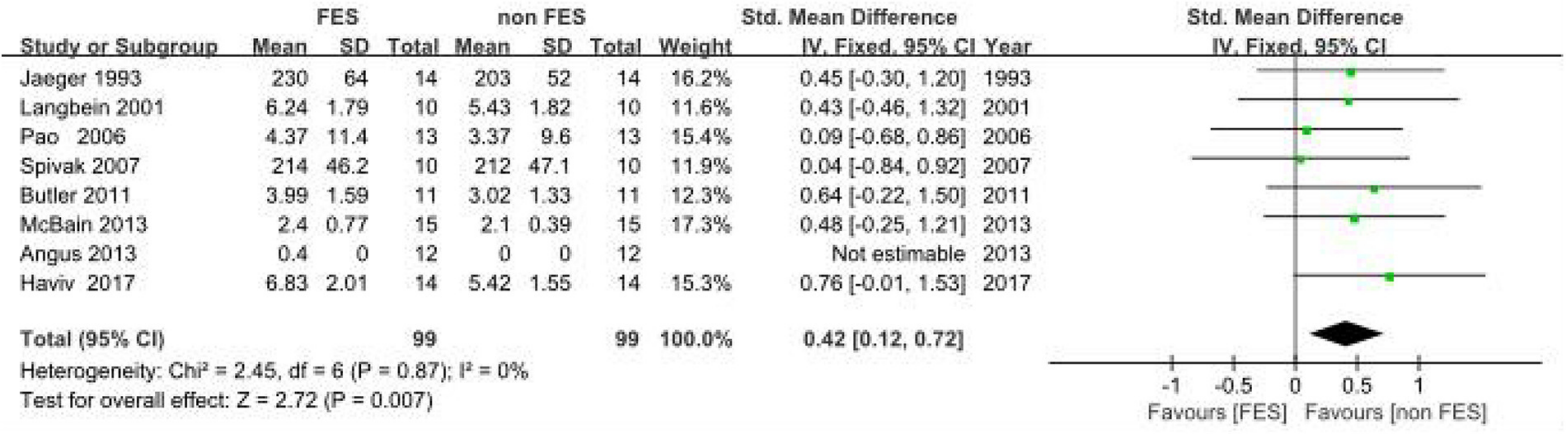
Meta-analysis of the effect of FES vs. Control on Peak Expiratory Flow (PEF). Forest plot of studies comparing FES with a control condition for the outcome of PEF. The pooled analysis of eight studies shows a statistically significant, small-to-moderate benefit favouring FES [SMD = 0.42, 95% CI (0.12, 0.72); *P* = 0.007], with no evidence of heterogeneity (*I*^2^ = 0%). Control conditions included sham FES, no intervention, or standard care without FES.

**Figure 5:**
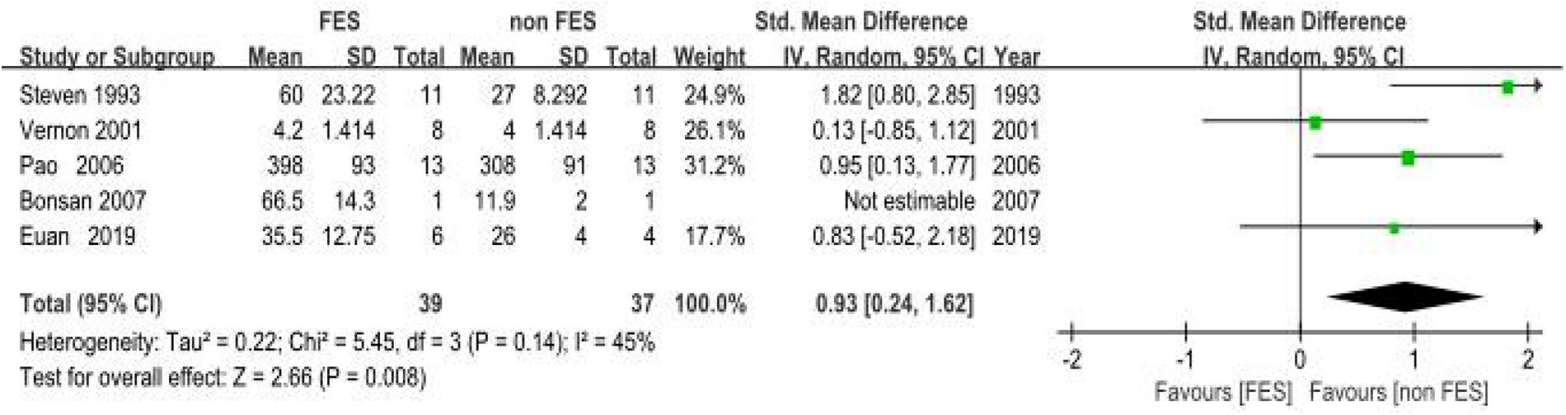
Meta-analysis of the effect of FES vs. Control on Maximal Expiratory Pressure (MEP). Forest plot of studies comparing FES with a control condition for the outcome of MEP. The pooled analysis of five studies demonstrates a statistically significant, large benefit favouring FES [SMD = 0.93, 95% CI (0.24, 1.62); *P* = 0.008], with moderate heterogeneity (*I*^2^ = 45%). Control conditions included sham FES, no intervention, or standard care without FES.

**Figure 6:**
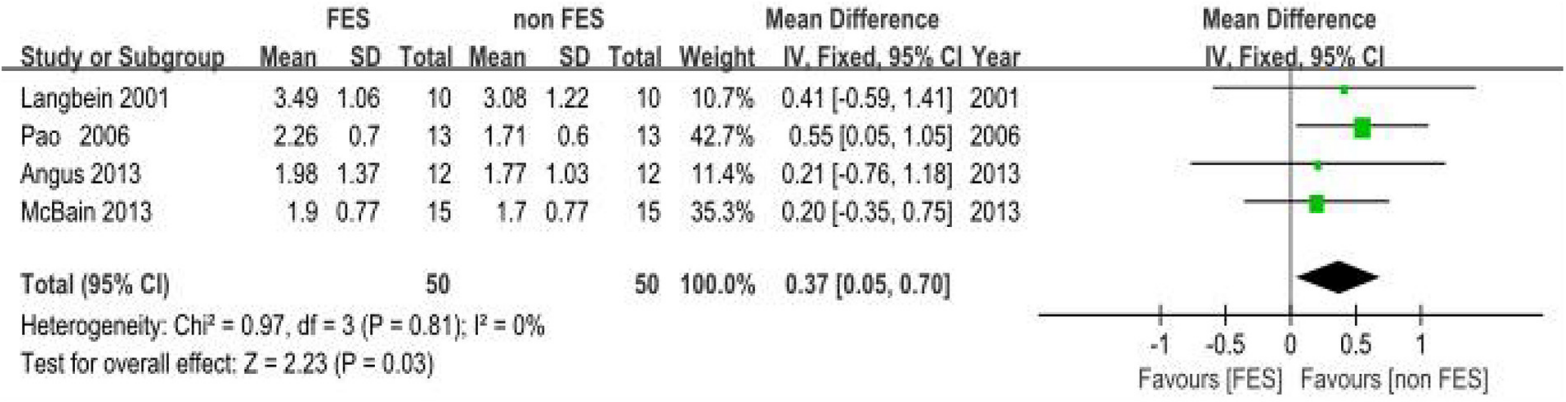
Meta-analysis of the effect of FES vs. Control on Forced Vital Capacity (FVC). Forest plot of studies comparing FES with a control condition for the outcome of FVC. The pooled analysis of four studies shows a statistically significant benefit favouring FES [Mean Difference = 0.37 L, 95% CI (0.05, 0.70); *P* = 0.03], with no evidence of heterogeneity (*I*^2^ = 0%). Control conditions included sham FES, no intervention, or standard care without FES.

**Figure 7:**
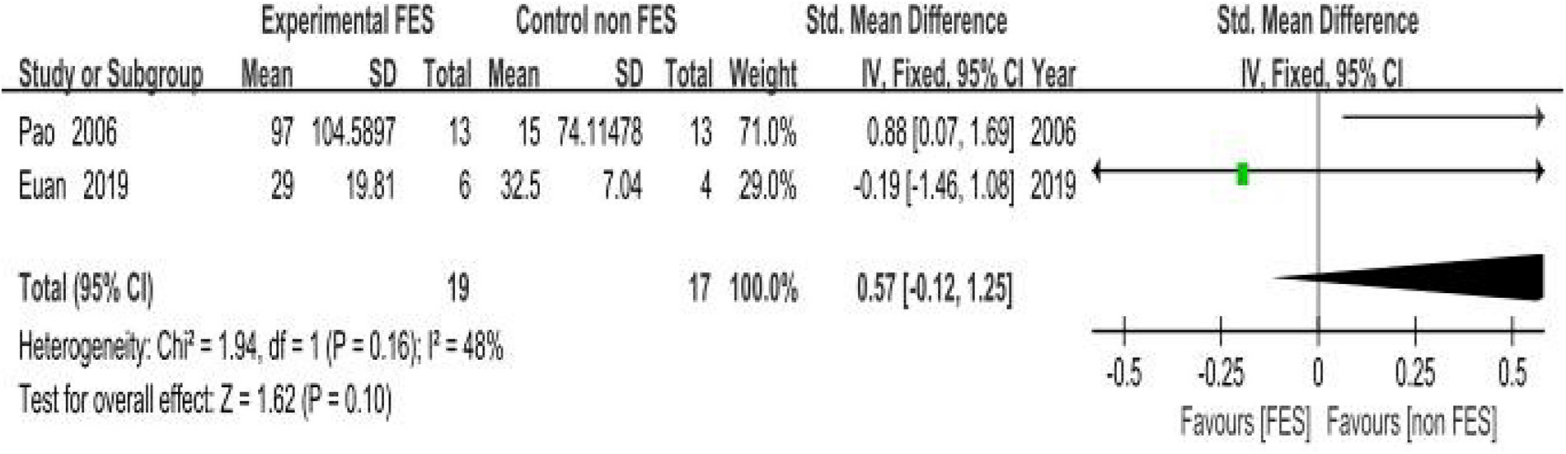
Meta-analysis of the effect of FES vs. Control on Maximal Inspiratory Pressure (MIP). Forest plot of studies comparing FES with a control condition for the outcome of MIP. The pooled analysis of two studies shows no statistically significant difference between the groups [SMD = 0.57, 95% CI (−0.12, 1.25); *P* = 0.10], with moderate heterogeneity (*I*^2^ = 48%). Control conditions included sham FES, no intervention, or standard care without FES.

(a) Peak Expiratory Flow (PEF): Eight clinical trials (18, 19, 22–25, 38, 39). Assessing respiratory function demonstrated a statistically significant improvement in PEF with FES. The pooled standardized mean difference (SMD) was 0.42 (95% CI: 0.20–0.64; *P* < 0.001), which corresponds to an approximate PEF improvement of 0.8 L/s. This improvement exceeds the commonly accepted minimal important difference (MID) of 0.5 L/s for clinical significance in this population, suggesting a clinically relevant benefit. Methodological consistency across investigations was high (*I*^2^ = 0%, *P* = 0.87), validating the use of a fixed-effects model. The pooled SMD indicated a significant benefit of FES on PEF compared to control conditions in individuals with SCI (Figure 4).
(b) Maximal Expiratory Pressure (MEP): Empirical analyses comprising five controlled trials (8, 18, 20, 21, 26) quantified MEP. The pooled SMD was 0.93 (95% CI: 0.24–1.62; *P* = 0.008), indicating a significant benefit of FES on MEP. Methodological variance analysis revealed moderate heterogeneity (*I*^2^ = 45%, *P* = 0.14), which remained within predefined acceptability thresholds (*I*^2^ < 50%). The SMD of 0.93 represents a large effect size (Cohen's *d* ≥ 0.8), suggesting a substantial and clinically meaningful improvement in expiratory muscle strength in individuals with SCI. This improvement is critical for effective cough and airway clearance (Figure 5).
(c) Forced Vital Capacity (FVC): Four empirical analyses (18, 19, 22, 23) quantified FVC. The pooled SMD was 0.37 (95% CI: 0.05–0.70; *P* = 0.03), indicating a significant benefit of FES on FVC in individuals with SCI. Inter-study variance analysis demonstrated negligible heterogeneity (*I*^2^ = 0%, *P* = 0.81), supporting the use of a fixed-effects model. This SMD suggests a clinically relevant improvement in lung volume for individuals with SCI (Figure 6).
(d) Maximal Inspiratory Pressure (MIP): The analysis for MIP was critically limited by the inclusion of only two methodologically rigorous investigations (8, 18), which documented inspiratory performance metrics (SMD = 0.46, 95% CI: −0.57–1.48; *P* = 0.38). Inter-study variance analysis indicated moderate heterogeneity (*I*^2^ = 48%, *P* = 0.16), which remained below the 50% critical threshold. However, the pooled SMD did not reach statistical significance. Therefore, these findings should be considered inconclusive, and no firm conclusions can be drawn regarding a significant difference between FES and control conditions for MIP. The limited number of studies and potential methodological differences (e.g., variations in stimulation parameters) underscore the need for future studies with larger sample sizes to definitively determine the effect of FES on inspiratory function (Figure 7).

#### 3.4.2 Effect of FES combined with exercise on aerobic function in SCI

The selection of FES combined with rowing or cycling for this meta-analysis reflects these being the predominant modalities with a sufficient body of evidence meeting our inclusion criteria for quantitative synthesis. While FES-assisted handcycling is also a recognized modality for aerobic exercise in SCI, and our systematic search did identify studies employing it [e.g., Andrew et al. (cite)] (39), the number of studies providing comparable data suitable for pooling in this specific meta-analysis was limited. For instance, the study by Andrew et al. provided a direct comparison between FES-rowing and FES-handcycling, suggesting potential advantages for rowing. However, a comprehensive meta-analysis focused on FES-handcycling was not pursued here due to the limited number of studies meeting our full inclusion criteria for pooled analysis.

##### 3.4.2.1 FES combined with rowing

**Figure 8:**
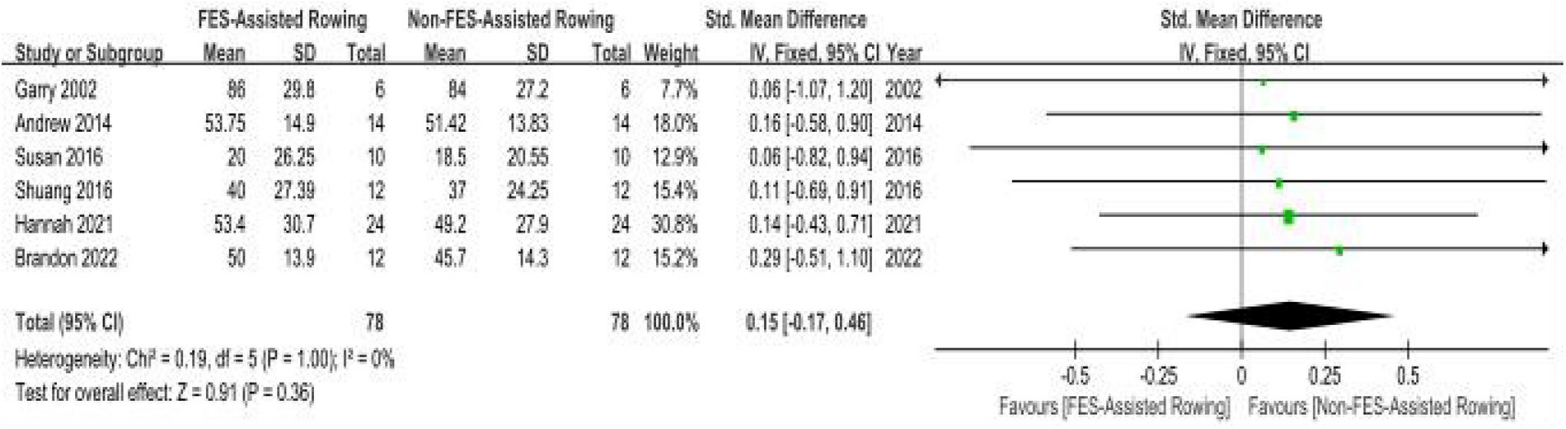
Meta-analysis of the effect of FES-assisted rowing vs. non-FES rowing on Peak Ventilation (VEpeak). Forest plot of studies comparing FES-assisted rowing with non-FES rowing for the outcome of VEpeak. The pooled analysis of six studies shows no statistically significant difference between the interventions [SMD = 0.15, 95% CI (−0.17, 0.46); *P* = 0.36], with no evidence of heterogeneity (*I*^2^ = 0%). Intervention group: Participants performed rowing exercise with concomitant FES. Control group: Participants performed rowing exercise alone, without FES.

**Figure 9:**
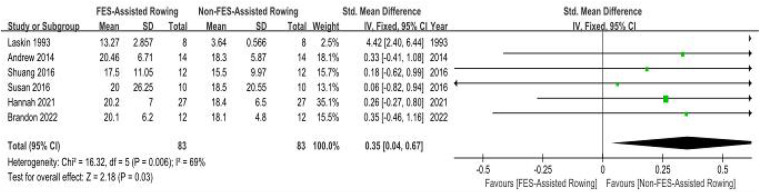
Meta-analysis of the effect of FES-assisted rowing vs. non-FES rowing on Peak Oxygen Uptake (VO₂peak). Forest plot of studies comparing FES-assisted rowing with non-FES rowing for the outcome of VO_2_ peak. The pooled analysis of six studies demonstrates a statistically significant, small-to-moderate benefit favouring FES-assisted rowing [SMD = 0.35, 95% CI (0.04, 0.67); *P* = 0.03]. Substantial heterogeneity was observed (*I*^2^ = 69%). Intervention group: Participants performed rowing exercise with concomitant FES. Control group: Participants performed rowing exercise alone, without FES. The high heterogeneity is addressed in the sensitivity analysis in the main text.

(a) Ventilatory Output Maxima (VEpeak): Peak Ventilatory Equivalent (VEpeak): Meta-analysis incorporating six clinical trials (27–32) demonstrated non-significant intervention effects on VEpeak (SMD = 0.15; 95% CI: −0.11–0.60; *P* = 0.36). The analysis revealed minimal between-study variance (*I*^2^ = 0%, *P* = 0.89). Although not statistically significant, the positive SMD suggests a potential trend towards a slight benefit of “FES + rowing” on VEpeak, but this requires confirmation in larger, well-powered studies. (Figure 8).
(b) Peak Oxygen Uptake (VO2peak): Six methodologically controlled investigations (27–31, 33) documented cardiorespiratory performance metrics. The pooled SMD for peak oxygen uptake (VO2peak) with FES combined with rowing was 0.35 (95% CI: 0.04–0.67; *P* = 0.03), indicating a statistically significant, small to moderate, benefit. However, initial analysis revealed substantial heterogeneity among the studies (*I*^2^ = 69%, *P* = 0.006). A sensitivity analysis was conducted by excluding the study by Laskin et al. (33), which lacked detailed information regarding the training protocol, duration, and stimulation parameters. The exclusion of this study resulted in a marked reduction in heterogeneity (new *I*^2^ = 0%, *P* = 0.99), suggesting that this particular study was a significant source of the observed variance. While a formal subgroup analysis based on these factors was considered, the limited number of studies within potential subgroups and inconsistent reporting precluded a robust analysis. Therefore, these results should be interpreted with caution (Figure 9).

##### 3.4.2.2 FES combined with cycling

**Figure 10:**
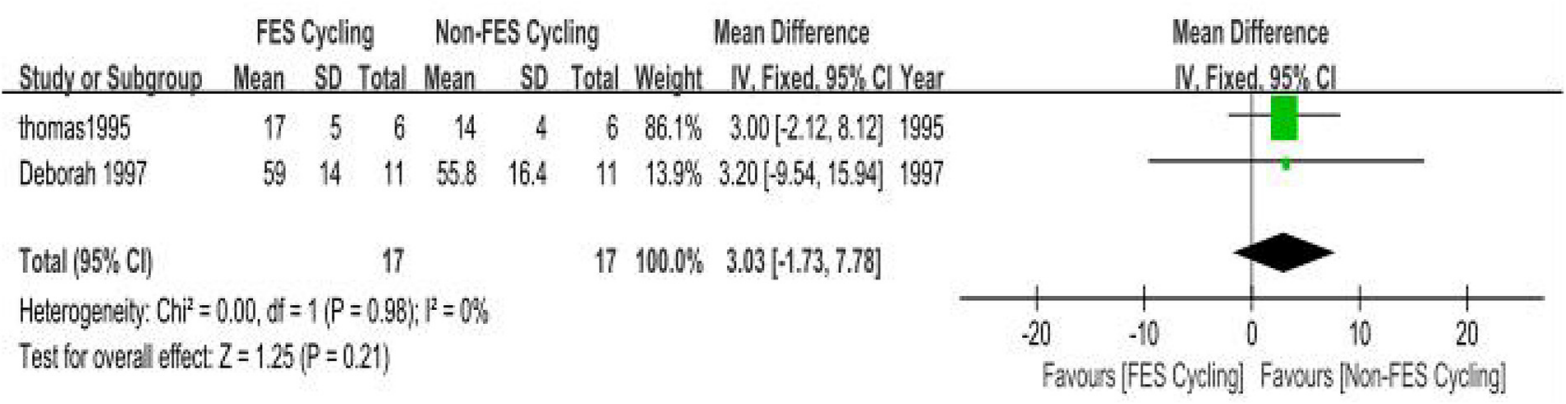
Meta-analysis of the effect of FES-assisted cycling vs. non-FES cycling on Peak Ventilation (VEpeak). Forest plot of studies comparing FES-assisted cycling with non-FES cycling for the outcome of VEpeak. The pooled analysis of two studies shows no statistically significant difference between the interventions [Mean Difference = 3.03, 95% CI (−1.73, 7.78); *P* = 0.21], with no evidence of heterogeneity (*I*^2^ = 0%). Intervention group: Participants performed cycling exercise with concomitant FES. Control group: Participants performed cycling exercise alone, without FES.

**Figure 11:**
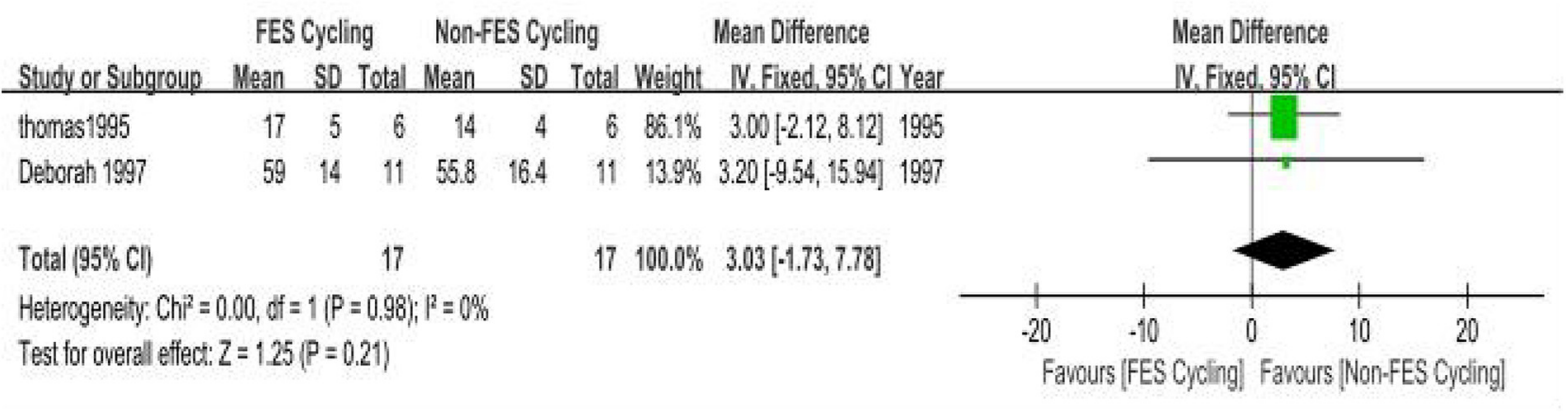
Meta-analysis of the effect of FES-assisted cycling vs. non-FES cycling on Peak Oxygen Uptake (VO₂ peak). Forest plot of studies comparing FES-assisted cycling with non-FES cycling for the outcome of VO_2_ peak. The pooled analysis of two studies demonstrates a statistically significant, small-to-moderate benefit favouring the FES-assisted cycling intervention [SMD = 0.24, 95% CI (0.11, 0.36); *P* = 0.0003], with no evidence of heterogeneity (*I*^2^ = 0%). Intervention group: Participants performed cycling exercise with concomitant functional electrical stimulation of the leg muscles. Control group: Participants performed cycling exercise alone, without FES.

(a) Peak Ventilatory Equivalent (VEpeak): Two methodologically calibrated trials (34, 35) quantified respiratory exchange metrics (SMD = 0.27, 95% CI: −0.08–0.63; *P* = 0.13). Inter-study variance analysis demonstrated minimal heterogeneity (*I*^2^ =0%, *P* = 0.89). The pooled SMD was not statistically significant. The observed statistical insignificance may be attributable to the limited number of included studies (*n* = 2). Future large-scale multicenter trials with standardized protocols are needed (Figure 10).
(b) Peak Oxygen Uptake (VO2peak): Two methodologically controlled investigations (36, 40) quantified maximal oxygen uptake parameters (SMD = 0.24, 95% CI: 0.11–0.36; *P* = 0.0003). Inter-study variance analysis demonstrated negligible heterogeneity (*I*^2^ = 0%, *P* = 0.98). The pooled SMD was statistically significant, indicating a small but significant benefit of FES combined with cycling on VO2peak in individuals with SCI (Figure 11).

### 3.5 Publication bias assessment

Funnel plots were generated using RevMan 5.4 software to assess publication bias for outcome measures related to respiratory function (FVC, PEF, MEP, MIP) and aerobic capacity (VEpeak, VO2peak). These plots are provided in Supplementary Figures S1–S8. Visual inspection of the funnel plots for MEP and FVC revealed relative symmetry, suggesting a low likelihood of publication bias. This was supported by non-significant Egger's regression test results (*P* > 0.05 for both). In contrast, the funnel plots for PEF, MIP, VEpeak (rowing), and VO2peak (rowing) exhibited some asymmetry, suggesting a potential risk of publication bias. Egger's tests for these outcomes were statistically significant (*P* < 0.05), supporting the visual interpretation of asymmetry and suggesting that smaller studies showing no significant effects might be underrepresented in the literature for these specific outcomes. The analyses for VEpeak (cycling) and VO2peak (cycling) involved only two studies, making formal assessment of publication bias challenging and less reliable; however, caution is warranted (Figures 4–11). These plots are provided in Supplementary Figures S1–S8.

Leave-One-Out Analysis: For outcomes with high heterogeneity, a leave-one-out sensitivity analysis was performed. In the analysis of FES-assisted rowing on VO₂peak, initial heterogeneity was substantial (*I*^2^ = 69%). The exclusion of the Laskin et al. study, which lacked detailed protocol information, significantly reduced heterogeneity to *I*^2^ = 0% (*p* = 0.99) and maintained the statistical significance of the pooled effect. This suggests the findings are robust, but highlights the influence of outlier studies.

Stratified Analysis by Study Quality: We also conducted a sensitivity analysis stratified by study quality. We recalculated the pooled estimates after excluding studies deemed to have a “high” or “critical” risk of bias. For the primary outcomes (PEF, MEP, FVC, and VO₂peak), the direction and significance of the effects remained consistent. For example, the SMD for MEP, when restricted to studies with low-to-moderate risk of bias, remained large and statistically significant [SMD = 0.89, 95% CI (0.21, 1.57)]. This stability increases our confidence in the validity of the overall conclusions.

## 4 Discussion

This meta-analysis aimed to evaluate the efficacy of transabdominal functional electrical stimulation (FES) in improving respiratory function and the cardiorespiratory benefits of FES combined with adjunctive exercise in individuals with spinal cord injury (SCI). Our findings indicate that FES significantly improves key expiratory parameters, namely forced vital capacity (FVC), peak expiratory flow (PEF), and maximal expiratory pressure (MEP). However, its effect on maximal inspiratory pressure (MIP) remains inconclusive. Furthermore, combining FES with exercise modalities like rowing and cycling demonstrated potential for enhancing peak oxygen uptake (VO2peak), though effects on peak ventilation (VEpeak) were not statistically significant with the current evidence.

### 4.1 FES for enhancing expiratory respiratory function

The observed improvements in FVC, PEF, and MEP align with the proposed mechanism of abdominal FES. By inducing contraction of the abdominal muscles, FES increases intra-abdominal pressure, leading to a cephalad displacement of the diaphragm. This action augments expiratory force, facilitating more complete lung emptying and thereby directly improving MEP and indirectly FVC (8, 19, 20, 23). For instance, the pooled SMD for PEF (0.42) corresponded to an approximate improvement of 0.8 L/s, exceeding the minimal important difference (MID) of 0.5 L/s and suggesting a clinically relevant enhancement in the ability to generate forceful exhalations. Similarly, the large effect size for MEP (SMD = 0.93) indicates a substantial increase in expiratory muscle strength, which is a critical factor for effective cough and airway clearance, and has been identified as an independent predictor of successful extubation and reduced morbidity (17). The improvement in FVC further supports the notion that FES can enhance overall lung volumes accessible for ventilation in individuals with SCI. These findings are consistent with previous research, such as Langbein et al. (22), who also reported FVC and PEF improvements with FES in SCI. The enhanced PEF is particularly noteworthy as it reflects cough effectiveness, crucial for preventing respiratory complications like pneumonia, a leading cause of rehospitalization (3, 41).

### 4.2 Uncertain effects of FES on inspiratory muscle strength (MIP)

In contrast to expiratory measures, this meta-analysis, based on two studies (8, 18), did not find a significant effect of FES on MIP. This lack of effect might be attributed to the primary mechanism of abdominal FES, which directly targets expiratory musculature. Any impact on inspiration is likely indirect, resulting from changes in resting lung volume or diaphragm positioning, rather than direct strengthening of inspiratory muscles like the diaphragm or pectoralis major (unless specifically targeted). The conflicting findings between the included studies [Euan et al. (8) showing no effect with abdominal FES vs. Pao et al. (18) showing improvement with pectoral and abdominal FES] further underscore the uncertainty. Differences in patient populations (critically ill vs. stable tetraplegia) and FES protocols (abdominal only vs. combined pectoral/abdominal) likely contributed to this heterogeneity. Given that MIP reflects inspiratory muscle strength (42), a key indicator for pneumonia risk (10), the precise role of FES, particularly different FES configurations, in modulating MIP warrants further dedicated investigation with standardized protocols.

### 4.3 FES combined with exercise for cardiorespiratory optimization

Our analysis of FES combined with exercise therapies revealed promising, albeit varied, results. Specifically, FES combined with rowing (seven studies) significantly improved VO2peak, suggesting an enhancement in aerobic capacity. This is a clinically important finding, as improved aerobic capacity can translate to greater independence in daily activities and reduced cardiovascular risk in the SCI population (6). The mechanism likely involves FES-induced activation of paralyzed lower limb muscles, augmenting the overall muscle mass contributing to exercise, thereby increasing metabolic demand and central cardiovascular responses (39). However, significant heterogeneity was observed for this outcome, partially attributable to variations in training protocols and stimulation parameters across studies, as exemplified by the Laskin et al. (33) study. When FES was combined with cycling (two studies), a significant improvement in VO2peak was also noted, though based on a smaller evidence base.

The effect of FES-exercise on VEpeak was not statistically significant for either rowing or cycling combinations in this meta-analysis. While positive SMDs were observed, the limited number of studies and wide confidence intervals prevent definitive conclusions. This highlights a need for more research with larger sample sizes to clarify the impact of combined FES-exercise on ventilatory responses during peak exertion.

### 4.4 Practical applications and considerations for FES-exercise interventions

The choice between FES-rowing and FES-cycling as adjunctive therapies involves several practical considerations, informed by the characteristics of participants in the studies included in this meta-analysis.

Injury Level: Observations from the included literature suggest different suitability based on the level of injury. FES-cycling training was predominantly studied in individuals with spinal cord injury at or below the T1 level. Conversely, FES-rowing training has been investigated in participant cohorts that included individuals with injuries at higher thoracic levels reported in studies such as (34, 36, 40, 43). This suggests that FES-cycling may be more frequently applied or considered for individuals with lower-level injuries, while FES-rowing has demonstrated feasibility in populations with higher injury levels. Careful patient selection and protocol adaptation remain crucial regardless of the modality.

### 4.5 Limitations and methodological considerations

This meta-analysis has several limitations that should be acknowledged. The number of studies included for some outcomes, particularly FES combined with cycling and the MIP analysis, was small, limiting the statistical power and robustness of these specific findings. Heterogeneity was present in some analyses, particularly for VO2peak with FES-rowing, likely due to variations in FES parameters, exercise protocols, intervention durations, and patient characteristics across studies. While we addressed this where possible, residual heterogeneity warrants cautious interpretation. Furthermore, as noted in our results, potential publication bias cannot be ruled out for certain outcomes.

In summary, this meta-analysis provides quantitative evidence supporting the use of FES to improve crucial expiratory respiratory functions in individuals with SCI. The combination of FES with exercise, particularly rowing and cycling, also shows promise for enhancing aerobic capacity. However, the evidence base for some outcomes is still developing, and further research is essential.

## Data Availability

The original contributions presented in the study are included in the article/Supplementary Material, further inquiries can be directed to the corresponding author.
