# Supplementary material for: Efficacy of functional electrical stimulation alone and as an adjunct to exercise for improving respiratory function and aerobic capacity in spinal cord injury: a systematic review and meta-analysis
Source: Front Rehabil Sci. 2025 Jul 31;6:1623752. doi: 10.3389/fresc.2025.1623752 (PMC12350409; doi:10.3389/fresc.2025.1623752)
Supplement: Supplementary file 1 [file Supplementaryfile1.doc]

| 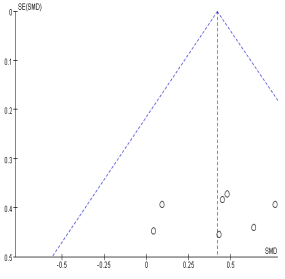  Caption: Morphological deviations within the meta-analysis visualization tool (funnel plot) indicate methodological distortions potentially attributable to selective research dissemination tendencies within the analyzed research corpus. |
| --- |
| S1. Risk of Publication Bias Assessment for PEF-Related Studies |

| 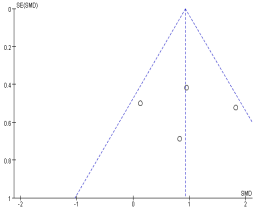  Caption: A symmetrical funnel plot suggests a lower risk of publication bias in the included studies. |
| --- |
| S2. Risk of Publication Bias Assessment for MEP-Related Studies |

| 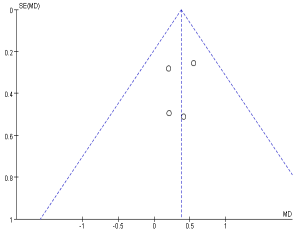C  caption: A symmetrical funnel plot suggests a lower risk of publication bias in the included studies. |
| --- |
| S3. Risk of Publication Bias Assessment for FVC-Related Studies |

| 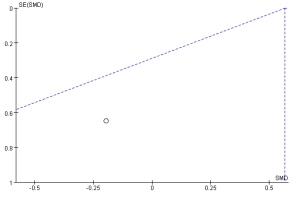  Caption: Morphological deviations within the meta-analysis visualization tool (funnel plot) indicate methodological distortions potentially attributable to selective research dissemination tendencies within the analyzed research corpus |
| --- |
| S4. Risk of Publication Bias Assessment for MIP-Related Studies |

| 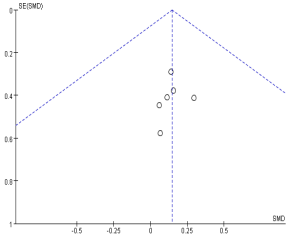  Caption: Morphological deviations within the meta-analysis visualization tool (funnel plot) indicate methodological distortions potentially attributable to selective research dissemination tendencies within the analyzed research corpus |
| --- |
| S5. Risk of Publication Bias Assessment for VEpeak-Related Studies |

| 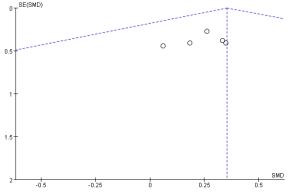  Caption: Morphological deviations within the meta-analysis visualization tool (funnel plot) indicate methodological distortions potentially attributable to selective research dissemination tendencies within the analyzed research corpus |
| --- |
| S6. Risk of Publication Bias Assessment for VO2peak-Related Studies |

| 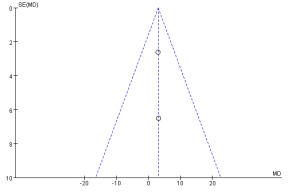  Caption: Morphological deviations within the meta-analysis visualization tool (funnel plot) indicate methodological distortions potentially attributable to selective research dissemination tendencies within the analyzed research corpus |
| --- |
| S7. Risk of Publication Bias Assessment for VEpeak-Related Studies |

| 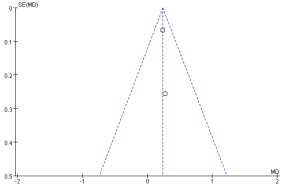  Caption: Morphological deviations within the meta-analysis visualization tool (funnel plot) indicate methodological distortions potentially attributable to selective research dissemination tendencies within the analyzed research corpus |
| --- |
| S8. Risk of Publication Bias Assessment for VO2peak-Related Studies |
